# Supplementary material for: Infection rate models for COVID-19: Model risk and public health news sentiment exposure adjustments
Source: PLoS One. 2021 Jun 28;16(6):e0253381. doi: 10.1371/journal.pone.0253381 (PMC8238235; doi:10.1371/journal.pone.0253381)
Supplement: S1 Appendix — (PDF) [file pone.0253381.s001.pdf]

Supplementary material for: 'Infection rate models for COVID-19: model risk and public health news sentiment exposure adjustments'

Ioannis Chalkiadakis*, Kevin Hongxuan Yan, Gareth W. Peters, Pavel V. Shevchenko

*

Version: June 15, 2021

Contents

Appendix 1

A Markov Chain Monte Carlo Convergence Diagnostics 2

B Sentiment index combining different news sources 3

C Pre-vaccination phase: in-sample fits for Spain, Italy and Japan 4

D Analysis Covering Pre- and Post-Vaccination Phases: in-sample fits for Spain, Italy and Japan 5

E Out-of-sample forecast study for Spain, Italy and Japan 7

F Sentiment exposure-adjusted in-sample fits 8

References 10

[0] *Corresponding author. E-mail: ic14@hw.ac.uk

A. Markov Chain Monte Carlo Convergence Diagnostics

To monitor the convergence for $k > 2$ chains of length $2n$ each, Gelman and Rubin (1992) proposed $\hat{R}$ which is defined as

$$\hat{R} = \frac{\hat{V}}{W} \cdot \frac{df}{df - 2},$$

where

$$\hat{V} = \frac{n-1}{n} W + \frac{k+1}{kn} B, \quad W = \sum_{i=1}^{k} \frac{s_i^2}{k}, \quad B = n \sum_{i=1}^{k} \frac{(\bar{\vartheta}_{i.} - \bar{\vartheta}_{..})^2}{k-1}, \quad df = \frac{2\hat{V}^2}{\hat{\mathrm{V}}\mathrm{ar}(\hat{V})},$$

$$\hat{\mathrm{V}}\mathrm{ar}(\hat{V}) = \left(\frac{n-1}{n}\right)^2 \frac{1}{k} \hat{\mathrm{V}}\mathrm{ar}(s_i^2) + \left(\frac{k+1}{kn}\right)^2 \frac{2}{k-1} B^2 +$$

$$2 \frac{(k+1)(n-1)}{kn^2} \cdot \frac{n}{k} [\hat{\mathrm{C}}\mathrm{ov}(s_i^2, \bar{\vartheta}_{i.}^2) - 2\bar{\vartheta}_{..} \hat{\mathrm{C}}\mathrm{ov}(s_i^2, \bar{\vartheta}_{i.})],$$

s_i is the within-chain variance and ϑ_{ij} is the j-th parameter in chain i, $\bar{\vartheta}_{i.}$ is the average over j, and $\bar{\vartheta}_{..}$ is the average over both i and j. If $\hat{R}$ is close to 1, the parameter ϑ has converged.

B. Sentiment index combining different news sources

For the sentiment index in this work, we considered a number of news sources that included worldwide circulation newspapers and national European and US institutions for disease control. We constructed separate sentiment indices for each of the selected news sources, and to construct a "global" index of sentiment magnitude we subsequently combined them into a single index, where each of the individual indices had a weighted contribution that was proportional to the "amount" of reporting. We measured the reporting amount in terms of article n-grams, which was the unit of text processing we employed in our study. As we describe in the main manuscript in Section 4.2.3, let $Y^{(s,j)}_{1:N^{s,j}}$, $s \in \{\text{health, economics, health} \cup \text{economics}\}$, $j \in \{$NYT, ECDC, USCDC, WHO, UNECE, Telegraph, Guardian, Reuters$\}$ be the text time-series corresponding to each of the selected sources of news reporting, where $N^{s,j}$ denotes the total number of n-grams of source j, with sentiment s. We can partition each time-series $Y^{s,j}_{1:N^{s,j}}$ into sets that contain observations that come from articles published on the same day: $Y^{(s,j)}_{1:n_1^{s,j}}, \ldots, Y^{(s,j)}_{1:n_T^{s,j}}$, where $n_t^{s,j} \geq 0$. The procedure that we followed next to combine the daily sets of the j news sources for days $t = 1, \ldots, T$ is described in Algorithm 1.

Input : a collection of entropy time-series $Y^{s,j}_{t=1:T}$, one per article source j, where each observation is the entropy of the distribution of token proportions per n-gram (Sec. 4.2.3 of the main manuscript), and each time-series has been constructed with the corresponding dictionary for a specific sentiment content s;
daily summary function $f(\cdot)$, e.g. median/IQR
Output : time-series Z^s_t summarising all available text sources
Initialisation : $Z^s = \emptyset$
for $t \leftarrow 1$ **to** T **do**
  for $j \leftarrow 1$ **to** N **do**
    `// summarise observations for a particular day` t `using` $f(\cdot)$
    $x_t = \{y^{s,j}_\tau : \tau = t\}$;
    $\tilde{y}^{s,j}_t = f(x_t)$;
    $w^{s,j}_t = |x_t|$;
  end
  $z^s_t = \sum_{j=1}^N \frac{w^{s,j}_t}{\sum_{k=1}^N w^{s,k}_t} \tilde{y}^{s,j}_t$;
  $Z^s \leftarrow Z^s \cup \{z^s_t\}$;
end

Algorithm 1: Construction of the joint entropy time-series.

C. Pre-vaccination phase: in-sample fits for Spain, Italy and Japan

In Figures A1 - A3 we plot the in-sample fits for Spain, Italy and Japan for the period of January 2020 to August 2020. We plot the baseline Model 2 and the best fitting model for each country, namely M12 for Spain and M7 for Italy - the baseline Model 2 also proved the best fitting model for Japan, which had a lower infection rate during the first wave of the pandemic. We note that the best fitting models for Italy and Spain exhibit their better fit to the infection data mostly by the narrower credible intervals.

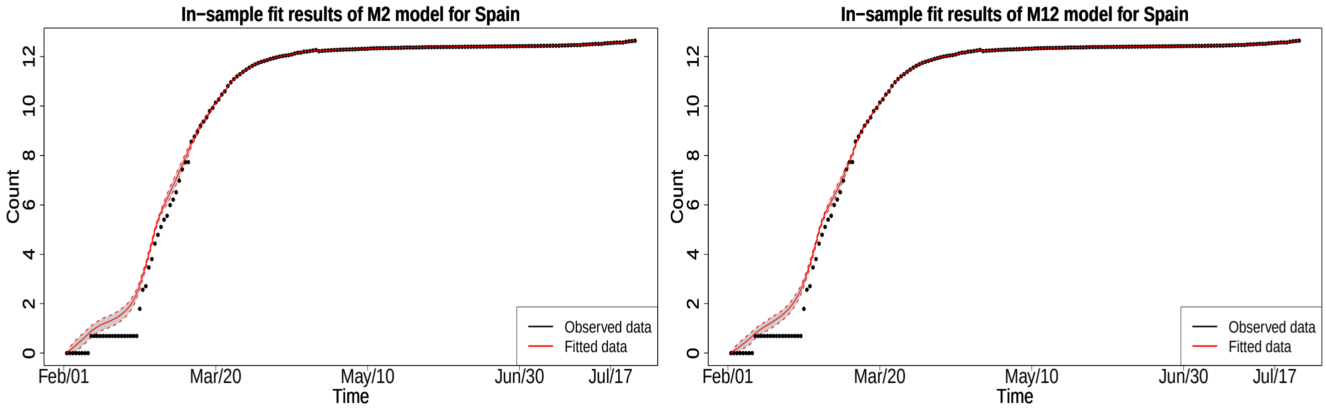

(a) **(b)**

Figure A1. In-sample fitted plot for Spain by Model 2 (baseline) and M12 (best)

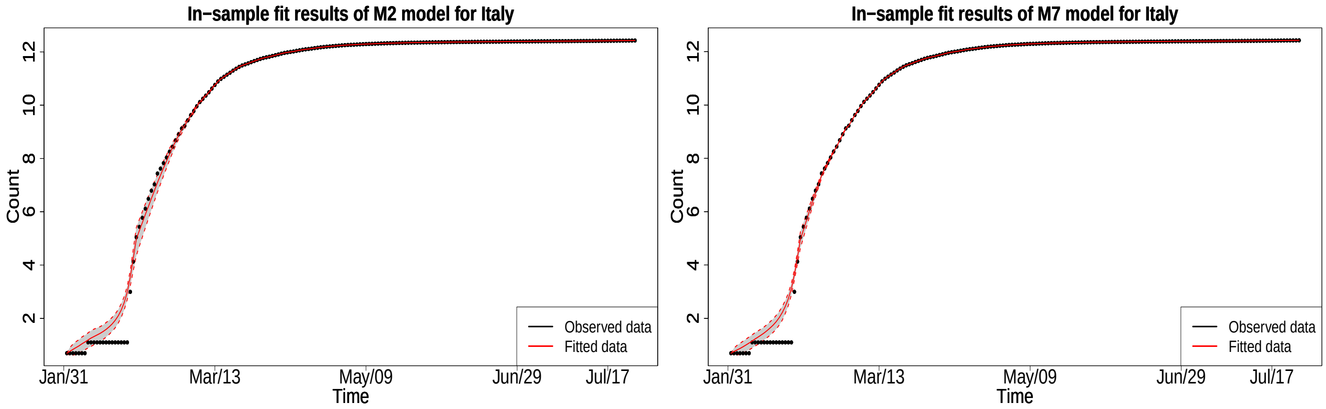

(a) **(b)**

Figure A2. In-sample fitted plot for Italy by Model 2 (baseline) and Model 7 (best)

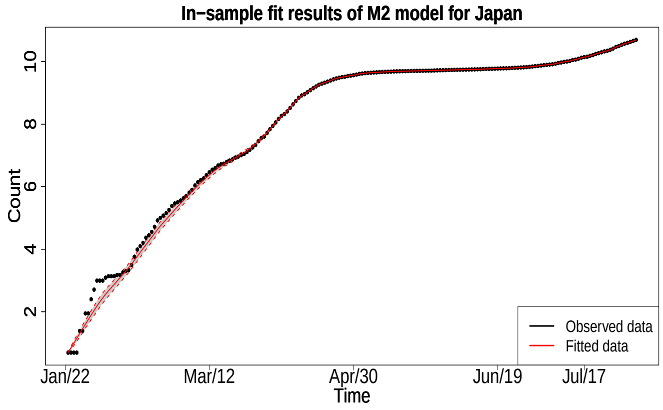

(a)

Figure A3. In-sample fitted plot for Japan by Model 2 (best)

D. Analysis Covering Pre- and Post-Vaccination Phases: in-sample fits for Spain, Italy and Japan

In this section we consider the full-length data analysis of January 2020 to January 2021, which spans both the pre-vaccine period and the period after the initiation of the vaccination program in many countries. We plot the traces of in-sample fits in Figures A4 - A6, and observe that for all of three countries M12 provided the best fit to the data. The difference in fitting quality is particularly evident in the fits for Spain and Italy where the baseline model fails to accommodate both the initiation of the pandemic and the period after the onset of the widespread community transmission. This is in accordance with our observation in the main manuscript that the epidemic in these countries exhibited a similar profile to the UK and Germany.

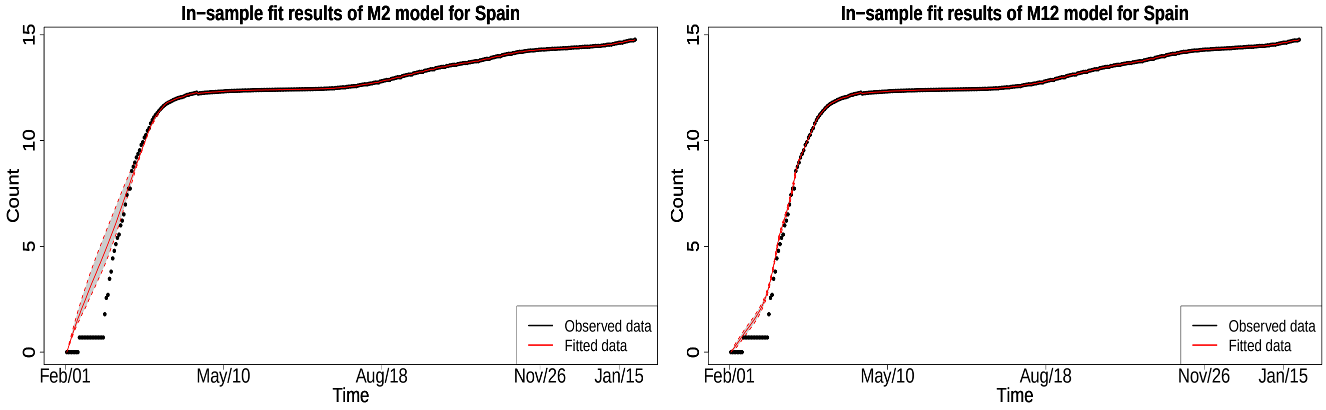

(a) **(b)**

Figure A4. In-sample fitted plot for Spain by Model 2 and Model 12 (January 2020 - January 2021)

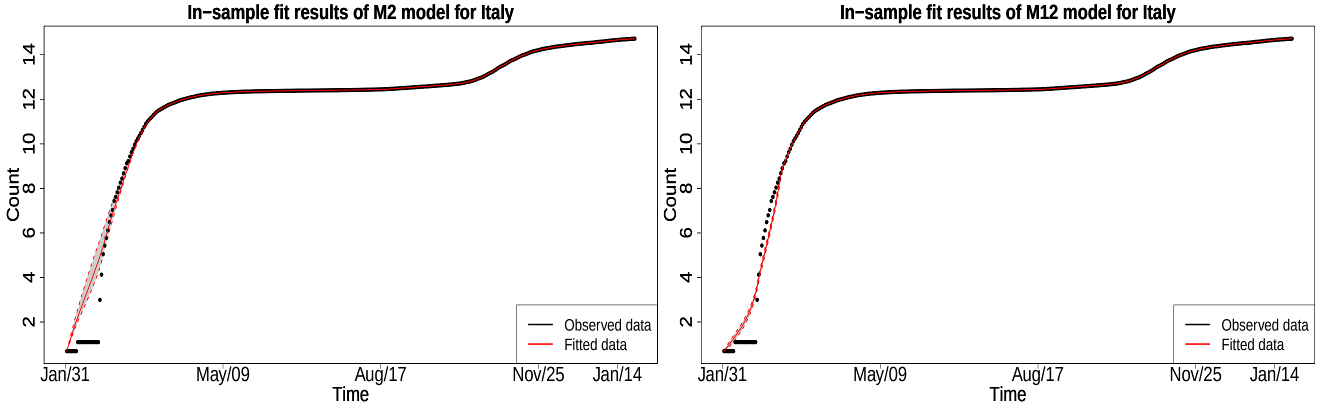

(a) **(b)**

Figure A5. In-sample fitted plot for Italy by Model 2 and Model 12 (January 2020 - January 2021)

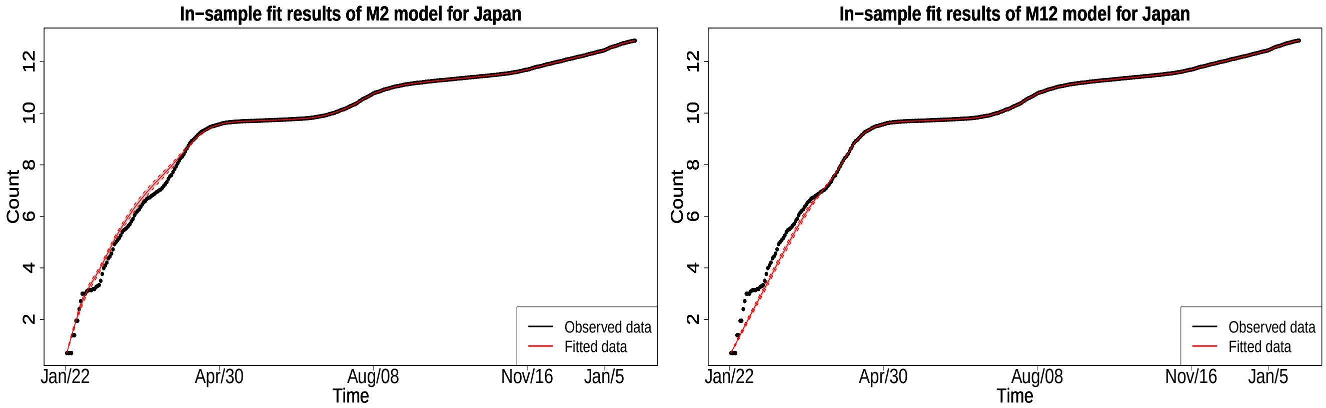

(a) **(b)**

Figure A6. In-sample fitted plot for Japan by Model 2 and Model 12 (January 2020 - January 2021)

E. Out-of-sample forecast study for Spain, Italy and Japan

We present the results of the out-of-sample forecast study for the three countries. We assessed the forecast for the baseline (M2) and best fitting model for each country (M12). In Figures A7 - A9 we observe the improvement in the best fitted model, in terms of mean point estimate for Spain and Italy. In the case of Japan, we note that M2 provides the best forecast performance, with M12 following closely, which justifies the slightly narrower credible interval we observe in Figure A9 (right panel).

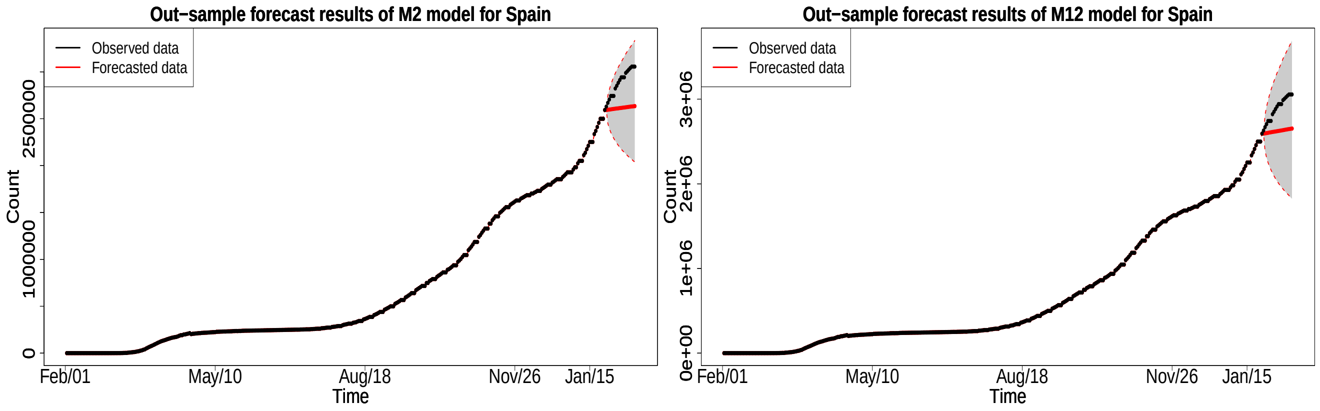

(a) **(b)**

Figure A7. Out-sample forecast plot for Spain by Model 2 and Model 12

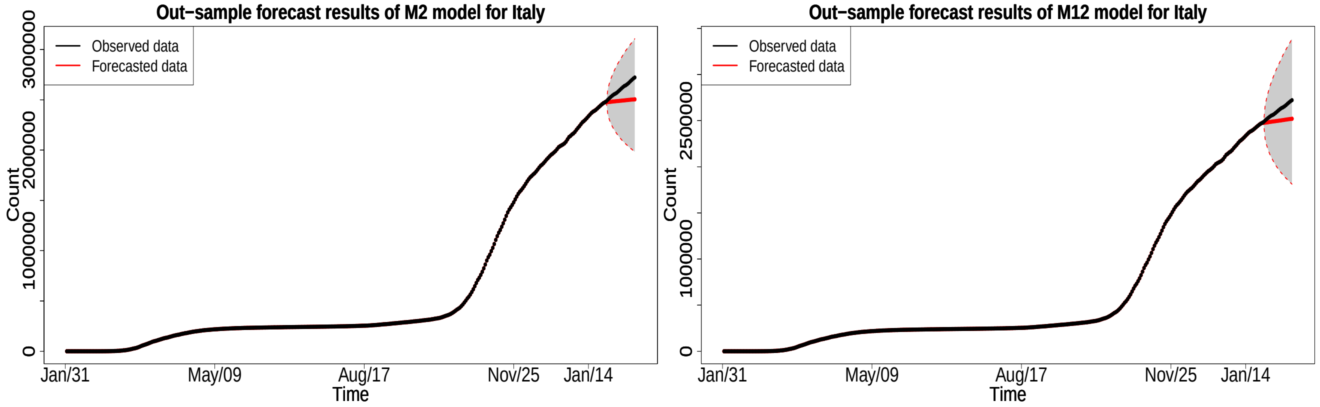

(a) **(b)**

Figure A8. Out-sample forecast plot for Italy by Model 2 and Model 12

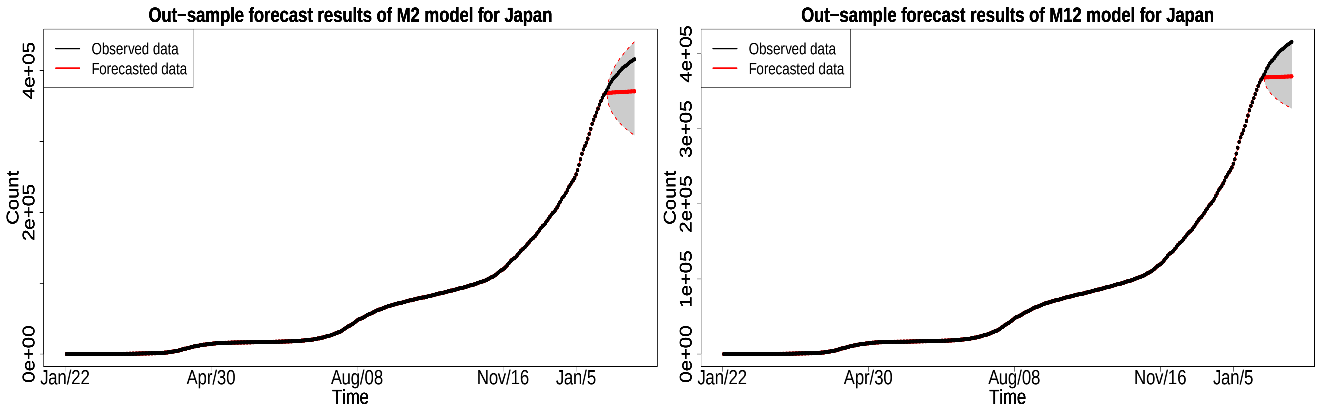

(a) **(b)**

Figure A9. Out-of-sample forecast plot for Japan by Model 2 and Model 12

F. Sentiment exposure-adjusted in-sample fits

In this section we present the outcome of the in-sample fitting exercise for the baseline model M2 at the start of the pandemic. As with the rest of the countries we documented in the main manuscript, we focus at the beginning of the pandemic due to the fact that at that time news reporting was very varying and people were more cautious towards the pandemic and more responsive to the related news. We observe Figures A10 - A12 and note that the left panels, which contain the trace plots for the models that included the sentiment exposure adjustment, clearly demonstrate the positive impact on the fit performance, both in terms of point estimates and narrower credible intervals. We quantify this improvement by computing the root mean squared error (RMSE) for the data in the figures and demonstrate the result in Table A1.

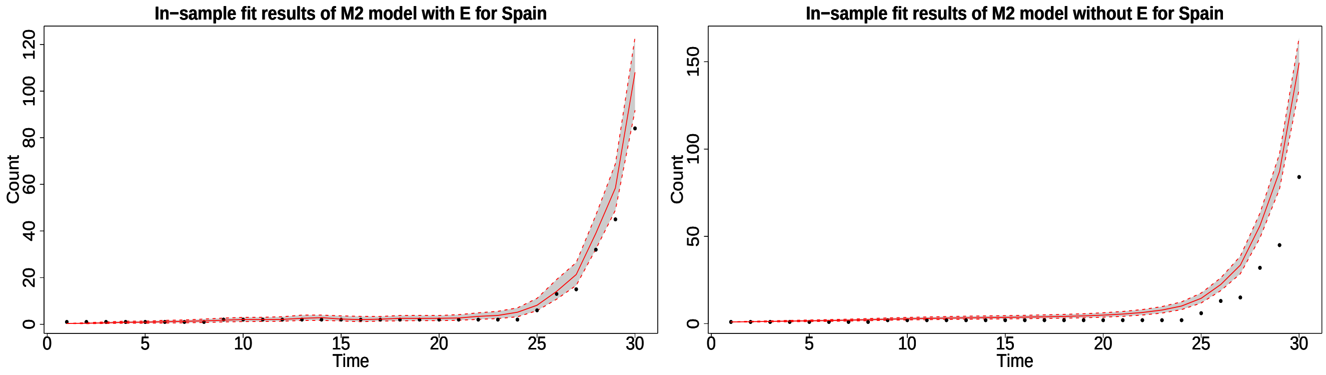

(a) **(b)**

Figure A10. In-sample fitting plot for Spain by Model 2 for the first month

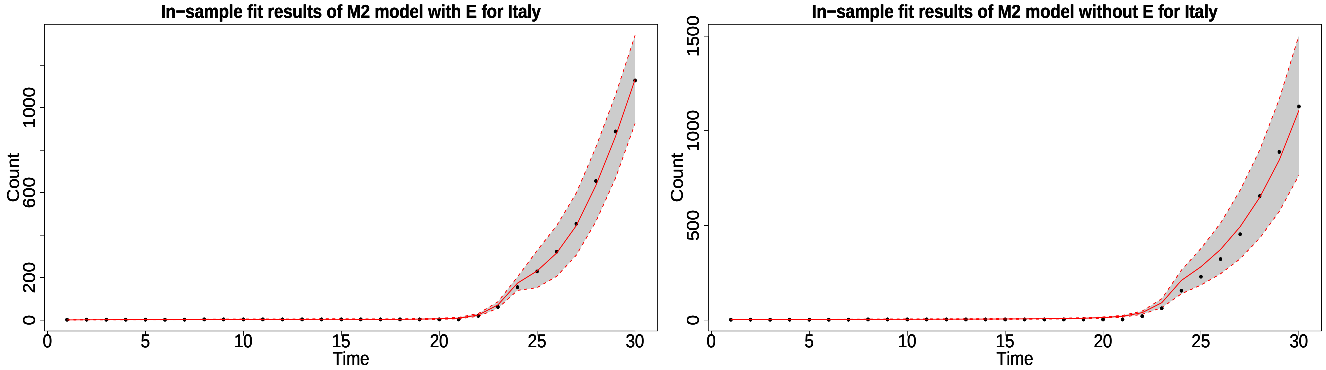

(a) (b)

Figure A11. In-sample fitting plot for Italy by Model 2 for the first month

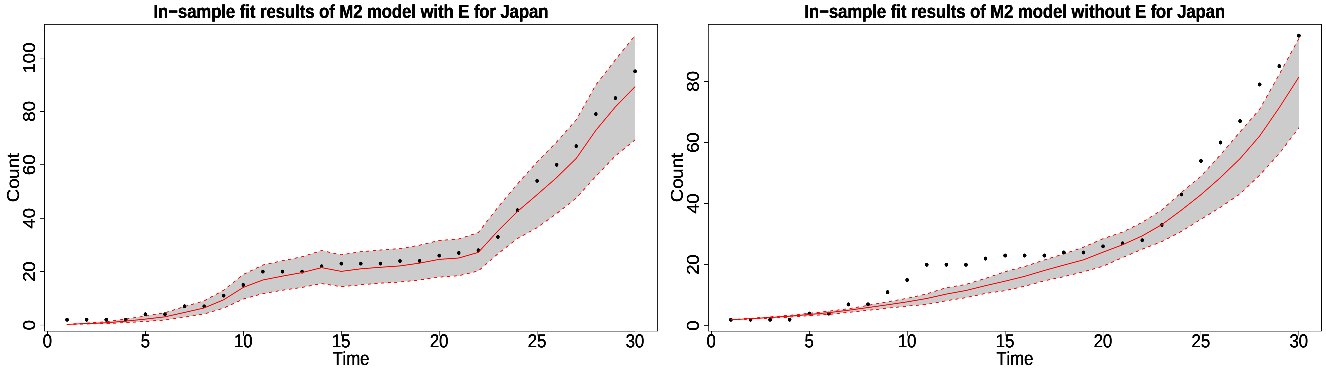

(a) (b)

Figure A12. In-sample fitting plot for Japan by Model 2 for the first month

Table A1. Root mean squared error (RMSE) for the in-sample fits for model M2 at the beginning of the pandemic, with and without the sentiment exposure adjustment.

**Country**	**In-sample fit with sentiment exposure (RMSE)**	**In-sample fit without sentiment exposure (RMSE)**
Japan	2.873	21.975
Italy	10.892	44.178
Spain	7.274	23.846

References

Gelman, A. and D. B. Rubin. 1992. Inference from iterative simulation using multiple sequences. *Statistical Science 7*(4), 457–472.
