# Supplementary figures and images for: Infection rate models for COVID-19: Model risk and public health news sentiment exposure adjustments
Source: PLoS One. 2021 Jun 28;16(6):e0253381. doi: 10.1371/journal.pone.0253381 (PMC8238235; doi:10.1371/journal.pone.0253381)

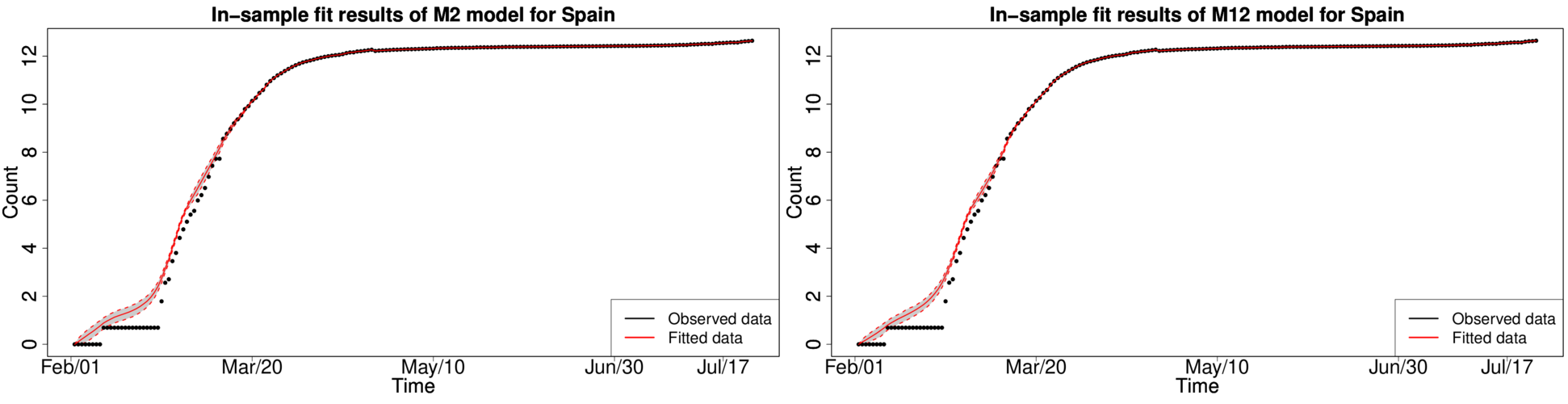

Supplement: S1 Fig — In-sample fitted plot (y-axis in log scale) for Spain by Model 2 (baseline, left) and Model 12 (best, right) (January 2020—August 2020). (TIF) [file pone.0253381.s002.tif]

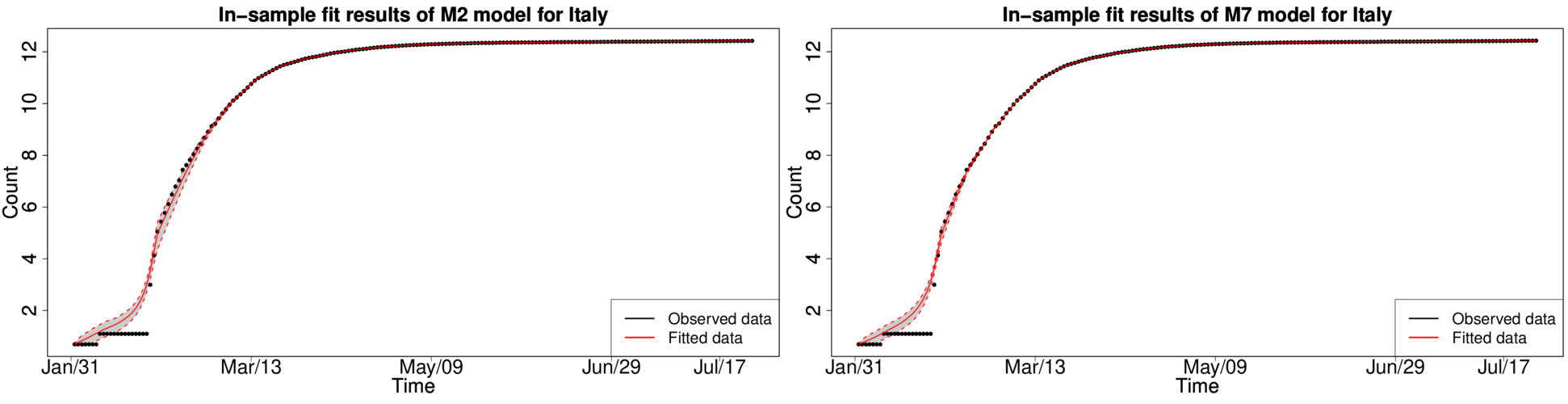

Supplement: S2 Fig — In-sample fitted plot (y-axis in log scale) for Italy by Model 2 (baseline, left) and Model 7 (best, right) (January 2020—August 2020). (TIF) [file pone.0253381.s003.tif]

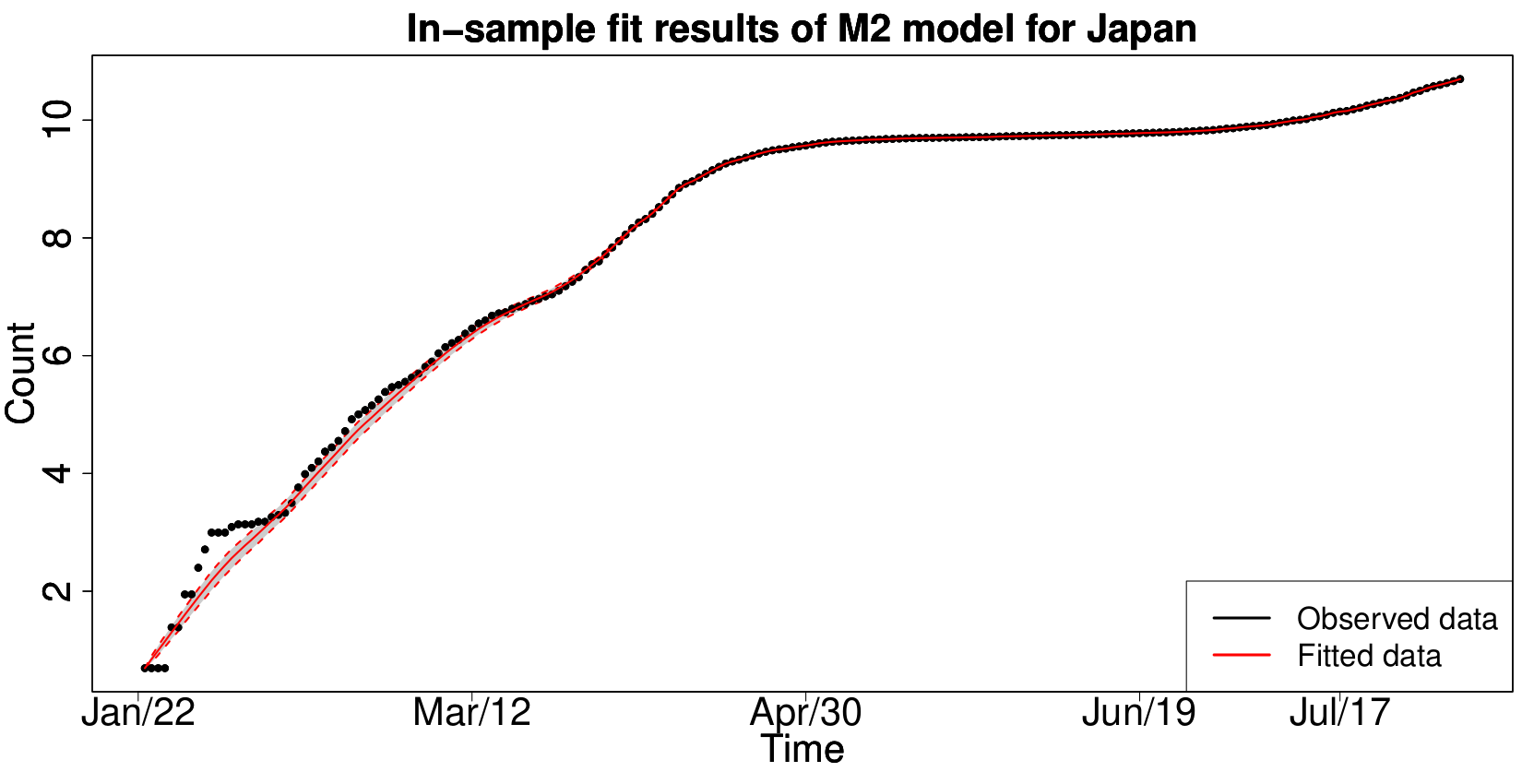

Supplement: S3 Fig — In-sample fitted plot (y-axis in log scale) for Japan by Model 2 (January 2020—August 2020). (TIF) [file pone.0253381.s004.tif]

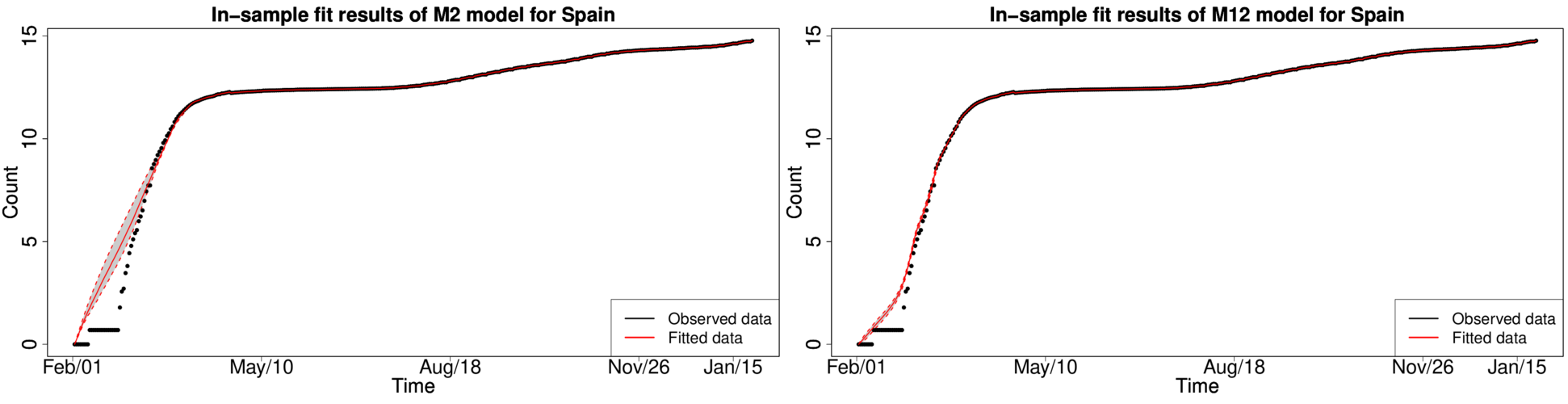

Supplement: S4 Fig — In-sample fitted plot (y-axis in log scale) for Spain by Model 2 (baseline, left) and Model 12 (best, right) (January 2020—January 2021). (TIF) [file pone.0253381.s005.tif]

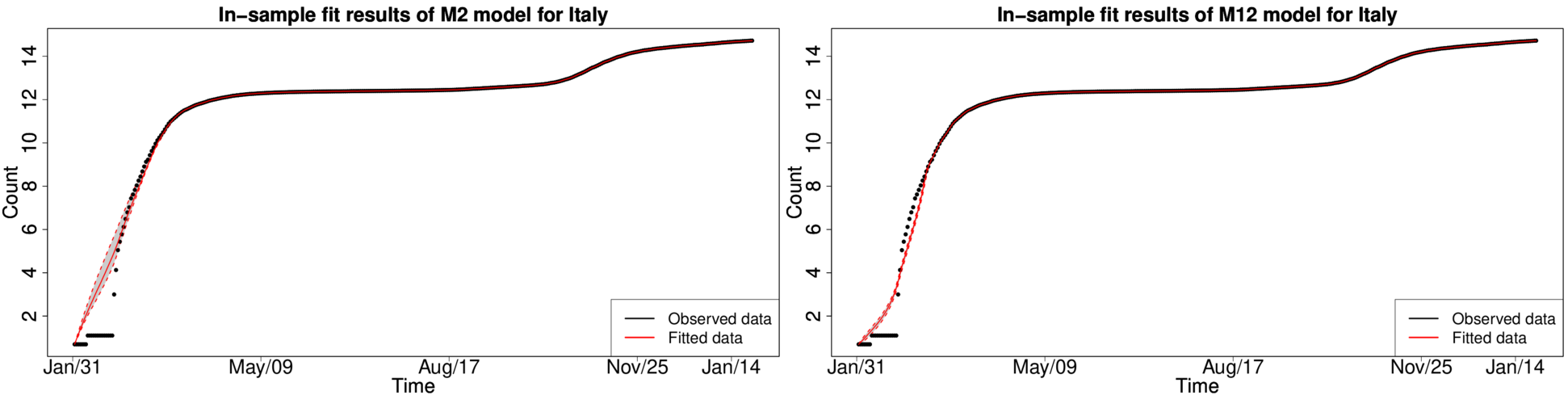

Supplement: S5 Fig — In-sample fitted plot (y-axis in log scale) for Italy by Model 2 (baseline, left) and Model 12 (best, right) (January 2020—January 2021). (TIF) [file pone.0253381.s006.tif]

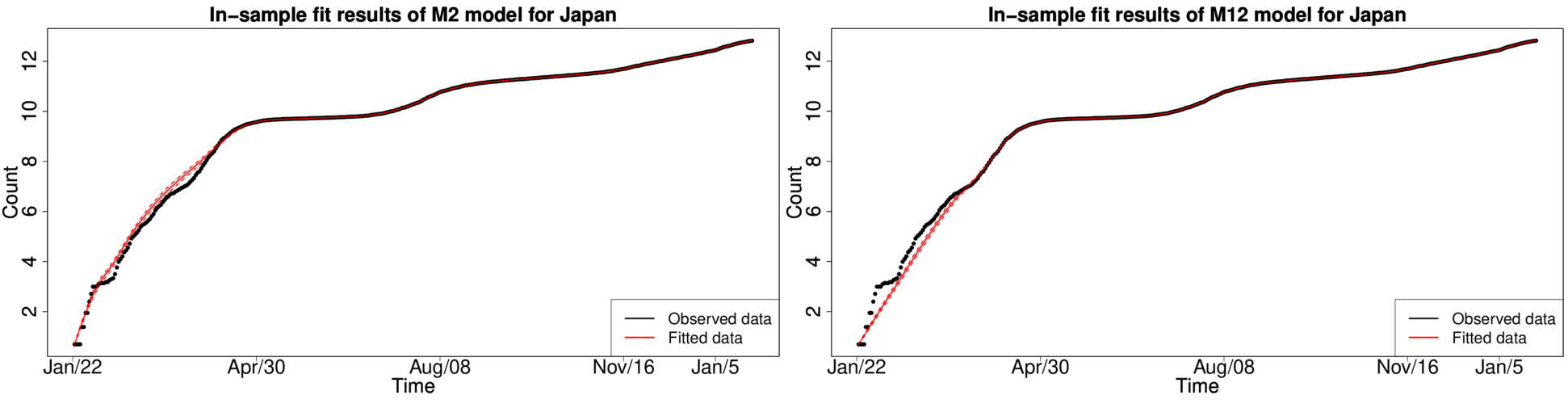

Supplement: S6 Fig — In-sample fitted plot (y-axis in log scale) for Japan by Model 2 (baseline, left) and Model 12 (best, right) (January 2020—January 2021). (TIF) [file pone.0253381.s007.tif]

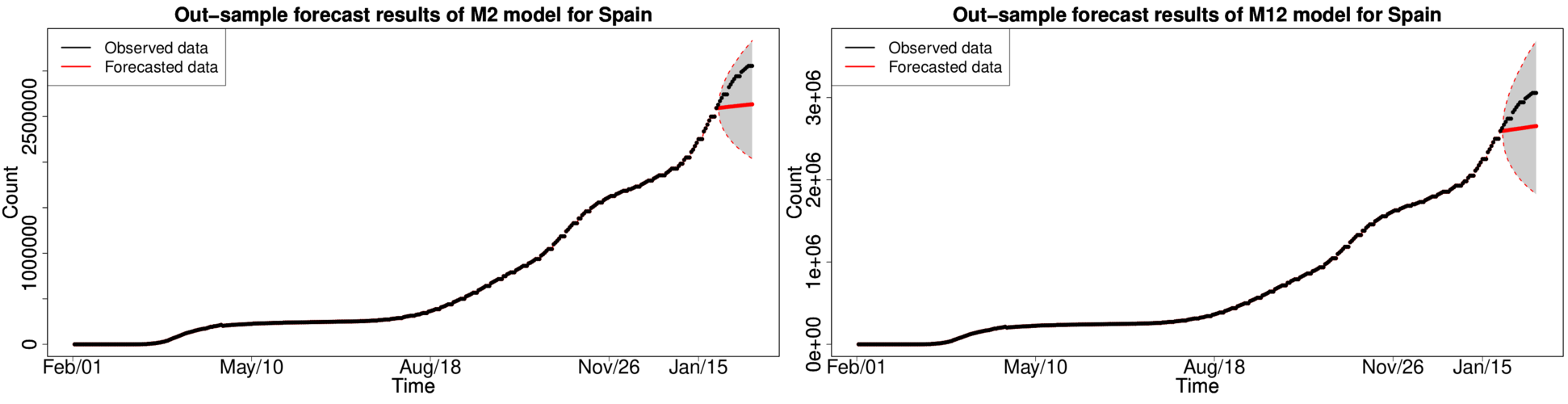

Supplement: S7 Fig — Out-of-sample forecast plot for Spain by Model 2 (left) and Model 12 (right). (TIF) [file pone.0253381.s008.tif]

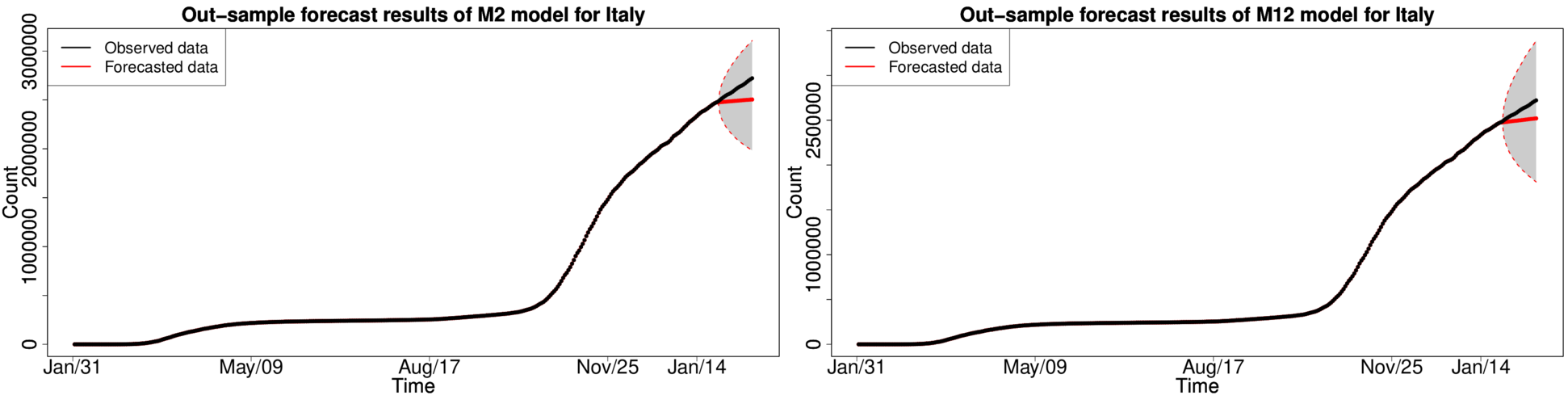

Supplement: S8 Fig — Out-of-sample forecast plot for Spain by Model 2 (left) and Model 12 (right). (TIF) [file pone.0253381.s009.tif]

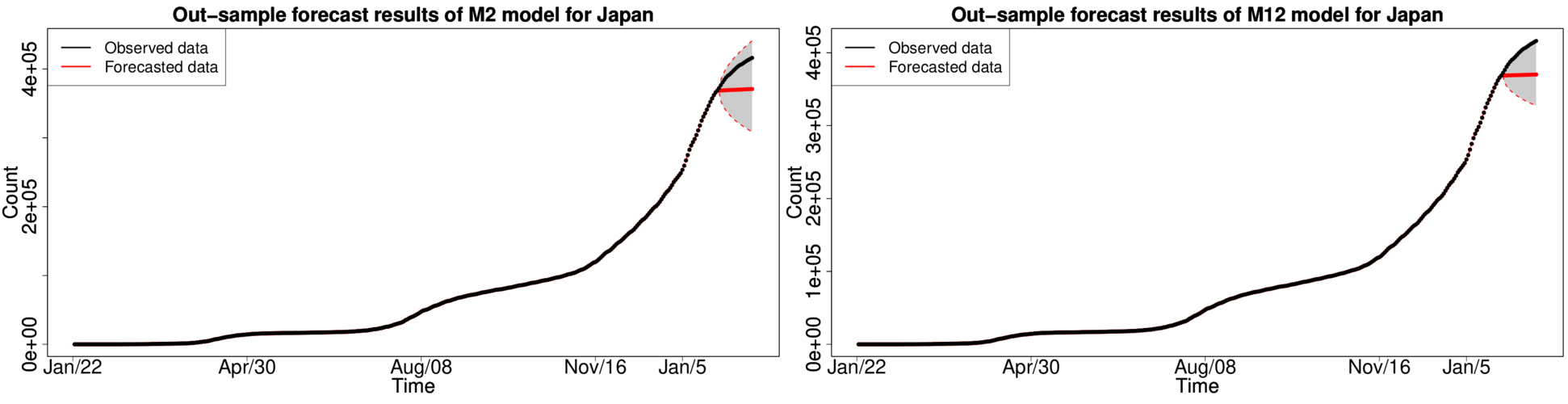

Supplement: S9 Fig — Out-of-sample forecast plot for Spain by Model 2 (left) and Model 12 (right). (TIF) [file pone.0253381.s010.tif]

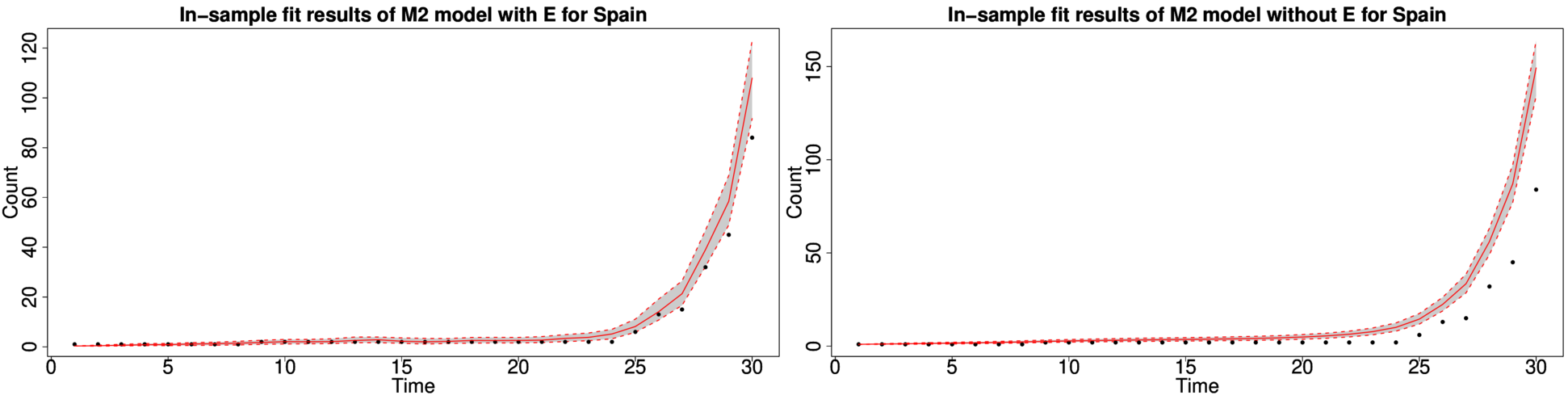

Supplement: S10 Fig — In-sample fitting plot for Spain by Model 2 for the first month with (left) and without (right) the sentiment exposure adjustment. (TIF) [file pone.0253381.s011.tif]

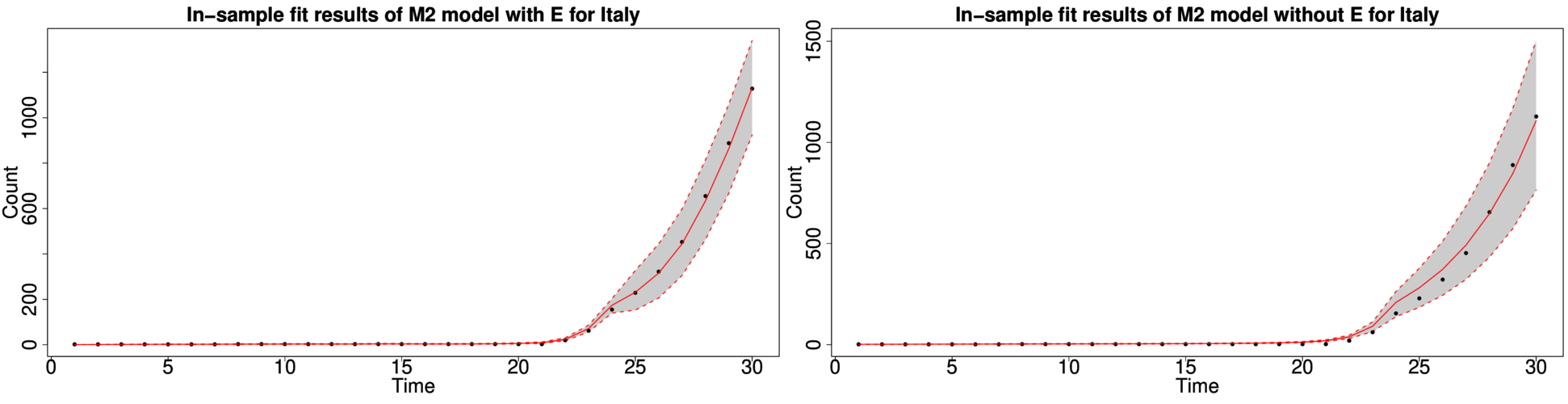

Supplement: S11 Fig — In-sample fitting plot for Italy by Model 2 for the first month with (left) and without (right) the sentiment exposure adjustment. (TIF) [file pone.0253381.s012.tif]

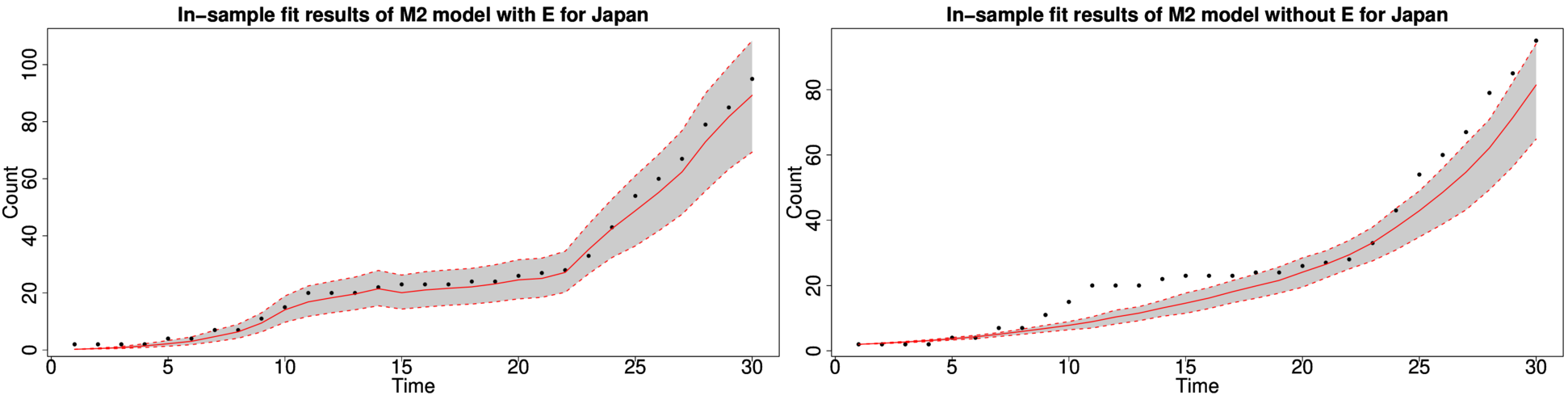

Supplement: S12 Fig — In-sample fitting plot for Japan by Model 2 for the first month with (left) and without (right) the sentiment exposure adjustment. (TIF) [file pone.0253381.s013.tif]
