# Supplementary material for: Infection rate models for COVID-19: Model risk and public health news sentiment exposure adjustments
Source: PLoS One. 2021 Jun 28;16(6):e0253381. doi: 10.1371/journal.pone.0253381 (PMC8238235; doi:10.1371/journal.pone.0253381)
Supplement: Attachment — Submitted filename: COVID19_Journal_Rebuttal_Letter_REVISION1.pdf [file pone.0253381.s015.pdf]

School of Mathematical and Computer Sciences

Heriot-Watt University
Edinburgh EH14 4AS, Scotland, UK

HERIOT WATT UNIVERSITY

Sunday, 30th May 2021

Dear Editors and Reviewers

Please find an itemised response letter outlining the modifications my co-authors and I have made to the manuscript:

'Infection rate models for COVID-19: model risk and public health news sentiment exposure adjustments'

by Ioannis Chalkiadakis, Dr. Hongxuan Yan, Prof. Gareth W. Peters, and Prof. Pavel V. Shevchenko

to respond to requests and suggestions by the Reviewers.

We begin by thanking the Reviewers and Editors for their time and suggestions to help enhance our manuscript and we are very pleased that all three reviewers and the associate editor found our work to be insightful and practically important for health policy making.

Furthermore, we are very pleased to see that all three Reviewers found our manuscript to be technically sound and that the data supported the presented conclusions. Given that we are big proponents of publicly available data and scientific source code for reproducibility in research, we are glad that all Reviewers have acknowledged our contribution in this respect.

We have updated several components to address the comments and questions raised by the Reviewers. Please find an itemised list of all questions and our response summaries in the remainder of this letter.

- Comments from Reviewers and Editors are in green
- Responses are in black

1. Editor Comment: Please ensure that your manuscript meets PLOS ONE's style requirements, including those for file naming. The PLOS ONE style templates can be found at `https://journals.plos.org/plosone/s/file?id=wjVg/PLOSOne_formatting_sample_main_body.pdf` and `https://journals.plos.org/plosone/s/file?id=ba62/PLOSOne_formatting_sample_title_authors_affiliations.pdf`

   Response: We thank the Editor for pointing this out. We have now made the necessary modifications to ensure that the manuscript complies to Plos One's style requirements. Please note that the figures in the manuscript are kept there for the convenience of the Reviewers. We have included the necessary figure references in the text in the requested positions, and we will also upload separate figure files in the required format once the manuscript is in publication stage.

2. Editor Comment: Please ensure that you refer to Figure 15 in your text as, if accepted, production will need this reference to link the reader to the figure.

   Response: We thank the Editor for spotting this. We are now referring to Figure 15 in section 6.3.1 'Out-of-sample forecast results for the UK, Germany and Australia':
   For the UK (Fig. 15) and Germany (Fig. 16), the model performance of M2 performs generally to the same level of accuracy as M7 and M12 in the forecast performance, despite the fact that in-sample fits of M2 for the full range of data are not as good as those obtained for M7 and M12 in-sample as demonstrated in (Table 6). We note that the in-sample fit superior Model 12 does provide slightly

narrower credible intervals than M2 for Germany, as we see in Fig. 16*, contrary to the UK, where the credible intervals appear slightly narrower for M2 (Fig.* 15*).*

3. Editor Comment: Please include captions for your Supporting Information files at the end of your manuscript, and update any in-text citations to match accordingly. Please see our Supporting Information guidelines for more information: `http://journals.plos.org/plosone/s/supporting-information`.

   Response: We thank the Editor for their comment. We have included the required references to the supporting information figures in a separate section in the text and will also upload the figures separately in the required format once the manuscript is in publication stage.

4. Reviewer 1 Comment: Abstract: It does not reflect clearly the setting and context. The seven countries and three approaches/considerations must be mentioned to inform the reader adequately.

   Response: We thank the Reviewer for this comment and incorporate the suggestion in our abstract so that we improve communication of the study context to the reader. We now clearly state that we study the United Kingdom (Wales, England, Scotland), Germany, Italy, Spain, Japan, Australia and the United States, for the period January 2020 to January 2021. Within the study period we identify two phases, namely the pre- and post-vaccine roll-out phases, the former covering January 2020 - August 2020, and together covering January 2020 - January 2021. For each period we state that we conducted the following studies: in-sample fitting, out-of-sample forecasting, and both of these with and without the sentiment exposure adjustment. We also state exactly the news sources we used to construct the sentiment index.

5. Reviewer 1 Comment: The countries selected and period selected should be clearly stated under a sub-heading like the research paper to appropriately inform the reader. At present it is lost in the text.

   Response: We thank the Reviewer for their comment on enhancing the presentation of our study so that it becomes more reader-friendly. We have now partitioned our results section in such a way as to reflect the different countries and study periods and we hope that this addresses this comment.

6. Reviewer 1 Comment: The source of the Language processing data - NYT has been considered. The NYT may be one of the richest source of news and public sentiment, it may not be adequate to reflect the public sentiment for other six countries. Why one leading source of news and public sentiment from the other countries were not considered? At least some comparative for the other countries and comparability between the news/public sentiments in NYT and other news sources should have been explored.

   Response: We thank the Reviewer for this comment, which we had also considered carefully when developing the paper. We have addressed this comment in two ways. Firstly, we outline the selection approach we adopted for news source selection. Then secondly, we explain how we have further significantly enhanced our analysis in response to this request.

   News Source Selection Criteria
   To begin with, when we set out to conduct our sentiment study we had set specific rules pertinent to the selection of news sources. These rules are outlined as follows below.
   First, we only considered news media publishing in the English language. Our sentiment index construction methodology currently works with English vocabulary and extending it to cover multiple languages was out of the scope of our current research work. In addition, we wanted to avoid the task of translation for two reasons: first, translating from the original language to English may affect sentiment which induces a certain risk in the construction of the sentiment index, and second, using automatic translation tools would also add the risk of the statistical model conducting the translation. This constraint would mean that we would not consider local media from Japan, Spain, Italy and Germany.
   Second, we only considered sources that included a dedicated section for COVID-19 coverage, so that we were certain that the articles we would process would be relevant. This would not be the case if the articles were not already categorised by the editors, and we had to identify ourselves that they cover aspects pertinent to COVID-19. This would induce an additional risk for the sentiment index construction. Note that we performed this selection process for articles coming from some of the Centres for Disease Control, however the scope of the reporting there is more limited and hence the articles are

easier to partition.
Third, we only considered news sources whose COVID-19 coverage was freely accessible to the public. The New York Times (NYT) is a major, worldwide news medium that satisfies all these requirements. It is part of the global collective effort to provide media and information to address the pandemic by offering a freely accessible curated collection of articles that cover many aspects of the pandemic. Furthermore, as we mention in the manuscript, the NYT attract a worldwide audience, which means that they provide reporting for the rest of the countries we considered, either from in-house journalists, or re-publishing reporting from global news agencies. Specifically, 19% of the NYT-published articles we processed were in fact coming from Reuters. We would also like to mention that 16% of the NYT subscribers come from outside the US (source: `https://letter.ly/new-york-times-readership-statistics/`), and this is excluding its non-subscribed readers, while Australia is the country with the fastest growing NYT readership.

New Source Sentiment Study Extensions
Furthermore, following the Reviewer's suggestion, we have significantly enhanced the sentiment study on a variety of additional news sources headquarted outside the U.S.A. In this regard, we constructed additional sentiment indexes from articles coming from major UK news media:

- The Guardian,
- The Telegraph; and
- Reuters global blog

the last of which offers an additional international influential news source. These additional sentiment studies involved collecting a large number of additional articles from these extra news sources for the same time period (January - August 2020).
We note that the coverage of the former is significant for European audiences (more than 50% of The Guardian's readership comes from all over Europe, source: `https://www.theguardian.com/advertising/audience1`).
A summary of the additional (in red) total number of articles collected and processed for sentiment construction is provided below (replicated in the paper in Table 1):

Source	Total #articles	Total #tokens	Total #sentences	Median of #tokens in article	Median of #sentences in article	Time period	Keywords
**European Centre for Disease Prevention and Control**	12	2,510	212	184	16	01/11/2019 - 06/08/2020	coronavirus, COVID19, nCoV, COVID-19, epidemic, pandemic, COVID-19, epidemic, pandemic, Italy, Spain, Greece
**The New York Times**	10,544	4,965,016	547,989	414	45	21/01/2020 - 06/08/2020	categorised by source
**United Nations Economic Commission for Europe**	52	17,508	1,287	304	23	23/03/2020 - 31/07/2020	categorised by source
**United States Centres for Disease Control and Prevention**	55	34,345	5,387	238	22	27/01/2020 - 30/07/2020	coronavirus, COVID-19
**World Health Organisation**	555	182,821	16,895	291	25	09/01/2020 - 06/08/2020	categorised by source
**The Telegraph**	11,918	3,289,002	343,226	247	24	23/01/2020 - 06/08/2020	categorised by source
**The Guardian**	13,719	4,960,699	515,643	335	32	23/01/2020 - 06/08/2020	categorised by source
**Reuters (blog)**	211	64,306	6,204	239	23	12/03/2020 - 06/08/2020	categorised by source

We were then able to demonstrate that our original index adequately represents the sentiment of the countries under study. To achieve this we have added to the manuscript, an additional table, Table 4, which shows the monthly robust median of the sentiment index for each of the reporting sources we additionally added (in red) into consideration in response to this Reviewer's request. (we also reproduce this table here for convenience):

News source	January	February	March	April	May	June	July	August
**ECDC**	2.313 ± 6.46E-02	2.646 ± 6.22E-02	2.267 ± 4.53E-02	-	2.422 ± 2.86E-02	2.296 ± 5.23E-02	-	-
**NYT**	2.265 ± 2.16E-02	2.043 ± 3.99E-03	1.987 ± 1.46E-03	1.979 ± 1.08E-03	1.976 ± 1.23E-03	1.996 ± 1.10E-02	2.012 ± 1.53E-03	2.016 ± 3.35E-04
**UNECE**	-	-	2.648 ± 1.62E-02	2.425 ± 4.63E-03	2.257 ± 1.64E-02	2.322 ± 9.81E-03	2.330 ± 9.78E-03	-
**USCDC**	2.380 ± 1.11E-02	2.221 ± 4.17E-03	2.180 ± 1.76E-02	2.088 ± 2.88E-02	2.060 ± 1.50E-03	2.109 ± 5.75E-03	2.166 ± 1.20E-02	-
**WHO**	2.684 ± 2.61E-01	2.401 ± 1.42E-02	2.174 ± 6.64E-02	2.123 ± 5.10E-03	2.098 ± 4.17E-03	2.072 ± 5.65E-03	2.090 ± 5.05E-03	2.104 ± 9.13E-03
**Telegraph**	2.186 ± 6.18E-03	2.047 ± 8.0E-04	2.125 ± 4.50E-04	2.138 ± 3.30E-04	2.139 ± 2.70E-04	2.146 ± 3.50E-04	2.137 ± 2.80E-04	2.154 ± 7.20E-04
**Guardian**	2.048 ± 5.45E-03	1.944 ± 1.14E-03	2.012 ± 5.50E-04	2.047 ± 4.50E-04	2.057 ± 5.20E-04	2.056 ± 6.0E-04	2.050 ± 5.70E-04	2.068 ± 1.40E-04
**Reuters**	-	-	2.402 ± 1.18E-02	2.229 ± 8.20E-03	2.180 ± 1.087E-02	2.136 ± 1.60E-02	2.112 ± 4.60E-02	2.145 ± 1.76E-02

We note that the fact that we did not observe differences in the results after adding the extra articles is consistent with the fact that the monthly entropy of the new articles (Telegraph, Guardian, Reuters blog) is similar to the entropy of the previously used news sources. This gave us strong confidence that the sentiment carried by the previously existing constructed index, and its influence as an exposure adjustment will still be present in each jurisdiction studied.

7. Reviewer 1 Comment: Discussion: Should be a separate segment and discuss the improvements/advantages and challenges compared to the real world setting observed and other models of prediction.

   Response: We appreciate the Reviewer's comment and we have now split our 'Results and Discussion' section into two separate sections, one for the results (Section 6) and one discussing and commenting on the results (Section 7).

8. Reviewer 1 Comment: Conclusion: A section on Conclusion should be mentioned to emphasize the findings from the research and future implications.

   Response: We thank the Reviewer for this suggestion. We have added a Conclusion section which emphasises the results and the associated implications of our findings as well as a brief mention of our plans for future work.

9. Reviewer 1 Comment: Table 4, 5, 6: The full form of DIC needs to be mentioned.

   Response: We appreciate the Reviewer pointing this out. Due to space it would be impractical to include the DIC in the caption of the tables, however we have improved its presentation in Equation 11, section 5.1 'Bayesian Model Selection and Forecast Performance', and referenced the equation in the captions of tables 5, 6 and 7 (updated numbering after the addition of an extra table).

10. Reviewer 2 Comment: Do the authors construct a single sentiment index based on the whole corpus collected from 5 different sources? This approach may bring some problems. For example, articles from New York Times make up the vast majority of the text sample. Although the paper argues that such a news source does attract a world-wide audience, at least in countries where English is amongst the official languages, I still think it is hard to be representative for other countries besides US. The reason is that the countermeasures taken by governments are mostly local based, thus people may not care about or be affected by anti-epidemic measures of other countries. Of course, the disease progress of other countries is also reported on New York Times, but people from other countries are most likely to pay attention to local newspapers. I suggest the authors to construct a separate sentiment index for US using articles from New York Times and United States Centres for Disease Control and Prevention, and for European countries using European Centre for Disease Prevention and Control and United Nations Economic Commission for Europe. Alternatively, the authors could separate article contents in the corpus for different countries and construct corresponding sentiment index.

    Response: We thank the Reviewer for this comment. We have indeed constructed a single sentiment index for the countries under study, and this was a decision based on the following reasons:

    i we could not use local media per country as we were restricted to English-language reporting only, due to the sentiment index construction framework,

    ii we did not want to partition the collection of articles into countries as that would introduce an additional risk of mis-classifying relevant reporting,

    iii we decided to combine all news sources as there was a significant imbalance in the number of articles that were available per month from the institutions and the Centres for Disease Control, and

    iv the counter-measures may have been local, as the Reviewer remarks, however we would argue that what was local was the timing and severity of the response, rather than the actual measures taken in the countries under study.

    In particular, all countries employed the following measures: quarantine and working from home directives, inter- and intra-country travel restrictions, masks, ban on gatherings and activities in closed spaces that involved people outside a basic 'bubble' of each person, and curfews. Furthermore, one could also identify the same reactions all over the world: some people adhered to the measures recognising the criticality of the circumstances, some people reluctantly adhered to the measures mainly due to fear of fines but actively resisted more the more restrictive the measures were getting, and finally some people completely ignored the measures not recognising the severity of the pandemic at all. Of course, it is also possible that people changed reaction group in the course of the pandemic, but it is safe to consider that these are representative of the majority of people's reactions.

    In addition, people will change their behaviour based on the underlying sentiment about the epidemic (fear, panic, uncertainty etc.), and governmental interventions taken to address the epidemic itself and

resulting repercussions. Such behavioural changes will feed back into the spreading mechanism of the epidemic and potentially will have a significant impact on its evolution. Consequently, they will also be the topic of news reporting covering the pandemic, and that will be the case for news media globally. Therefore, we believe that a global news medium such as the New York Times, together with more local reporting from the Centres for Disease Control adequately captures the society's sentiment. As we also mentioned in our response to Comment 6, Table 4 shows that the sentiment information of the public as captured by the extra news sources is not considerably different to the sentiment captured by NYT and the Centres for Disease Control.

11. Reviewer 2 Comment: The results of this paper demonstrate that including a sentiment index significantly enhanced the model fit. I think this additional explanatory power of the news sentiment should be quantified to make the conclusion more convincing. Furthermore, does sentiment index improve out-sample forecasts in the study?

    Response: We thank the Reviewer for the valuable comment. We had indeed evaluated the out-of-sample performance, which is conditional on the available amount of training data, of the sentiment-adjusted model for a one-month future window.
    Our findings were rather interesting in this regard. We found that the most significant effects measured statistically in model fit MSE and the forecast accuracy metrics employed, arose from the inclusion of the sentiment exposure adjustments in early periods of the pandemic. However as the pandemic progressed, the importance of the model stochastic trend structure was far more pronounced compared to the marginal contributions of the sentiment exposure adjustment. In other words, the important part in the forecast exercise was the choice of the model for the growth rate curve, rather than the exposure adjustment after the first few months of the pandemic's evolution.
    We believe the justification for this may be related to a saturation in public messaging and clear repeated guidance, less uncertainty regarding the disease and less initial fear that arose from mixed public messaging in the first 4-6 months of the pandemic. This logic is entirely consistent with our findings.
    As we demonstrate in the manuscript, the sentiment adjustment was substantial for the in-sample fit early on in the pandemic outbreak. We have performed additional analysis to further emphasise this as requested. We have now included an additional table in the paper, Table 9 (included below for convenience):

**Country**	**In-sample fit with sentiment exposure (RMSE)**	**In-sample fit without sentiment exposure (RMSE)**
US	0.903	7.262
Germany	1.489	5.221
Australia	1.593	7.366
United Kingdom	2.942	17.184

    These results clearly demonstrate the improvement that sentiment exposure adjustments made to model performance in the initial highly uncertain period of the study (4-6 months into the pandemic).
    As mentioned, this is also in accordance with the argument that we are making, namely that people were much more receptive to the protection measures at the beginning of the pandemic, due to the uncertainty surrounding much of the reporting at the onset of the novel coronavirus spreading. Nevertheless, as the pandemic evolved and the counter-measures were relaxed and re-instated multiple times, the public became more accustomed to them and the effect of the news reporting on the society got saturated.

    We also appreciate the Reviewer's suggestion to quantify the improvement that the sentiment adjustment contributed to the model fit. For this purpose we added Table 9 in the manuscript, which as we mentioned above, demonstrates the root mean squared error of the in-sample fit evaluated for the baseline model with and without the sentiment exposure adjustment.

12. Reviewer 2 Comment: It is advised that the authors discuss the limitations of the study and give the future research work in conclusion part.

    Response: We thank the Reviewer for this suggestion. We have added a Conclusion section that discusses limitations and future research plans.

13. Reviewer 2 Comment: Some equations are too long and hard to read. Try to refine them using some

notations.

Response: We appreciate the Reviewer's comment and we acknowledge that the formulation we have developed for the construction of the text time-series may be considered hard to read. Therefore, we have tried to improve its presentation by introducing clarifying notation for the n-grams and the indicator function we use. We hope that equations 4-9 will be easier to read now and address the Reviewer's suggestion. Regarding the Bayesian formulation, we note that the posterior predictive distribution is quite standard and we feel an attempt to simplify it by introducing extra notation would have the opposite result of what we try to achieve. Furthermore, the long second line of equation 3 for the complete likelihood is just the expansion of the already introduced notation in the first line for the Generalised Poisson and Gaussian distributions, and therefore we believe that explicitly showing all the algebra to arrive at that expression would consume space without it being necessary as it is easy to derive.

14. Reviewer 2 Comment: I suggest the authors provide depict figure or descriptive statistics of the sentiment index.

    Response: We thank the Reviewer for their suggestion. We have now added Table 4 which shows the median sentiment with a 95% confidence interval per reporting medium, per month. We believe this adequately describes the index, as well as the sentiment content of the different news media, and we hope that this sufficiently addresses the Reviewer's comment.

15. Reviewer 2 Comment: There are some grammatical errors and/or instances of poorly worded/constructed sentences. Please carefully check the manuscript/abstract, and refine the language where necessary.

    Response: We appreciate the Reviewer's comment regarding the readability of the manuscript. We checked the manuscript multiple times, corrected any grammatical errors we had missed in the first submission, and also improved some of our expressions to make them easier to follow. We have also passed the manuscript through automatic grammar checkers (Grammarly).

16. Reviewer 3 Comment: The authors stated that they used the sentiment analysis technique, however, they did not classify sentiment polarity. As it reads now, the sentiment analysis in this paper just sounds like topic or keyword analysis using dictionaries. Is this indeed the case? I suggest that the authors give more details about how they construct the dictionary.

    Response: We appreciate the Reviewer's comment and took it into serious consideration as one of the novelties of the paper is to demonstrate the construction of the particular sentiment index, and we want to make sure we communicate it clearly to the reader.
    To begin with, it is not exactly true that the sentiment analysis we present is plain topic or keyword analysis, like a Latent Dirichlet Allocation topic model or a clustering approach would do. We identify two steps in our approach to constructing the sentiment index.
    The first is the construction of the text time-series. This is based on the idea of counting the occurrence of English words, which we could broadly refer to as keywords since we have focused on vocabulary of specific topics (epidemics, politics, business, psychology) in addition to a general English vocabulary. However, the novelty is first, in constructing the dictionary, which we detail in the manuscript, and second, in formulating the construction into a time-series, by gradually modifying the occurrence counts as more text is fed into the system. As we have now added in the manuscript, there are few approaches that specifically construct a text time-series from news articles with the intention to be used in statistical time-series regression models as we do. Most approaches to date have been simple, such as replacing each word with the number of characters that comprise it, or constructing binary time-series from text via applying simple logical functions such as checking presence of particular tokens, or capturing keywords in short social media, e.g. Twitter, texts.
    The second step is the construction of the sentiment index from the text time-series by quantifying the entropy of distribution of the n-grams, as described in equation 9 in the manuscript. This is different to most approaches that either use keyword counting of specific sentiment-expressive words, or some complex model, e.g. a deep neural network, which induces high computation costs and renders the results difficult - if able at all - to interpret. At this point we would also like to remark that in another work of ours we have specifically studied and demonstrated how our approach captures different information from popular NLP sentiment models based on BERT (Bert: Pre-training of deep bidirectional transformers for language understanding, by J Devlin, MW Chang, K Lee, K Toutanova,

2019) or VADER (Vader: A parsimonious rule-based model for sentiment analysis of social media text, by C Hutto, E Gilbert, 2014). The Reviewers are welcome to see our studies in an appendix we have uploaded in the following repository: `https://github.com/ichalkiad/cryptogpcausality/tree/main/appendix`.
Following the advice of the Reviewer we have also enhanced section 4.2.1 of the manuscript where we discuss the construction of the dictionary and specifically point out its strengths over common practice. We hope that the response and modifications in the manuscript improve the presentation of our work and thank the Reviewer for their attention.

We believe we have addressed carefully and with great effort all the valuable suggestions and comments of the reviewers and we hope this has improved the paper for this journal submission.

We thank the reviewers and editors for their time and consideration.

Yours sincerely,
Ioannis Chalkiadakis
