# Supplementary material for: Infection rate models for COVID-19: Model risk and public health news sentiment exposure adjustments
Source: PLoS One. 2021 Jun 28;16(6):e0253381. doi: 10.1371/journal.pone.0253381 (PMC8238235; doi:10.1371/journal.pone.0253381)
Supplement: Attachment — Submitted filename: Referee Report-PONE-D-21-11059.docx [file pone.0253381.s014.docx]

Referee Report: Infection rate models for COVID-19: model risk and public health news sentiment exposure adjustments

This paper models the temporal evolution of national level infection counts for a variety of countries by exploring a variety of stochastic population growth models. The authors characterize model risk mathematically, and develop an exposure adjustment to the force of infection comprised of a purposed built sentiment index constructed from a variety of authoritative public health reporting. They find that exposure adjustments that incorporate sentiment are better able to calibrate to early stages of infection spread in all countries under study.

The paper makes for a smooth read, while the methodology and empirical results are well described. The research is insightful for health policy making. My comments and suggestions are presented below.

1. GENERAL COMMENTS

- Do the authors construct a single sentiment index based on the whole corpus collected from 5 different sources? This approach may bring some problems. For example, articles from New York Times make up the vast majority of the text sample. Although the paper argues that such a news source does attract a world-wide audience, at least in countries where English is amongst the official languages, I still think it is hard to be representative for other countries besides US. The reason is that the countermeasures taken by governments are mostly local based, thus people may not care about or be affected by anti-epidemic measures of other countries. Of course, the disease progress of other countries is also reported on New York Times, but people from other countries are most likely to pay attention to local newspapers.

I suggest the authors to construct a separate sentiment index for US using articles from *New York Times* and *United States Centers for Disease Control and Prevention*, and for European countries using *European Centre for Disease Prevention and Control* and *United Nations Economic Commission for Europe*. Alternatively, the authors could separate article contents in the corpus for different countries and construct corresponding sentiment index.

- The results of this paper demonstrate that including a sentiment index significantly enhanced the model fit. I think this additional explanatory power of the news sentiment should be quantified to make the conclusion more convincing. Furthermore, does sentiment index improve out-sample forecasts in the study?
- It is advised that the authors discuss the limitations of the study and give the future research work in conclusion part.

2. SPECIFIC COMMENTS

- Some equations are too long and hard to read. Try to refine them using some notations.
- I suggest the authors provide depict figure or descriptive statistics of the sentiment index.
- There are some grammatical errors and/or instances of poorly worded/constructed sentences. Please carefully check the manuscript/abstract, and refine the language where necessary.
